# Supplementary material for: Repetitive Trans Spinal Magnetic Stimulation Improves Functional Recovery and Tissue Repair in Contusive and Penetrating Spinal Cord Injury Models in Rats
Source: Biomedicines. 2021 Dec 3;9(12):1827. doi: 10.3390/biomedicines9121827 (PMC8698720; doi:10.3390/biomedicines9121827)

Figure S1: rTSMS treatment does not have effect on sensitive recovery after penetrating SCI in rats. Quantification of hindpaw withdrawal thermal latency at (A) 15 days, (B) 30 days and (C) 60 days after SCI. Quantifications are expressed as average + SD. N=14 animals per group at 14 and 30 days after SCI and N=10 animals per SCI group and N=11 animals per Stm group at 60 days after SCI. Dashed lines correspond to the baseline parameters obtained with non-injured animals. Statistical evaluations were based on Mann-Whitney test.

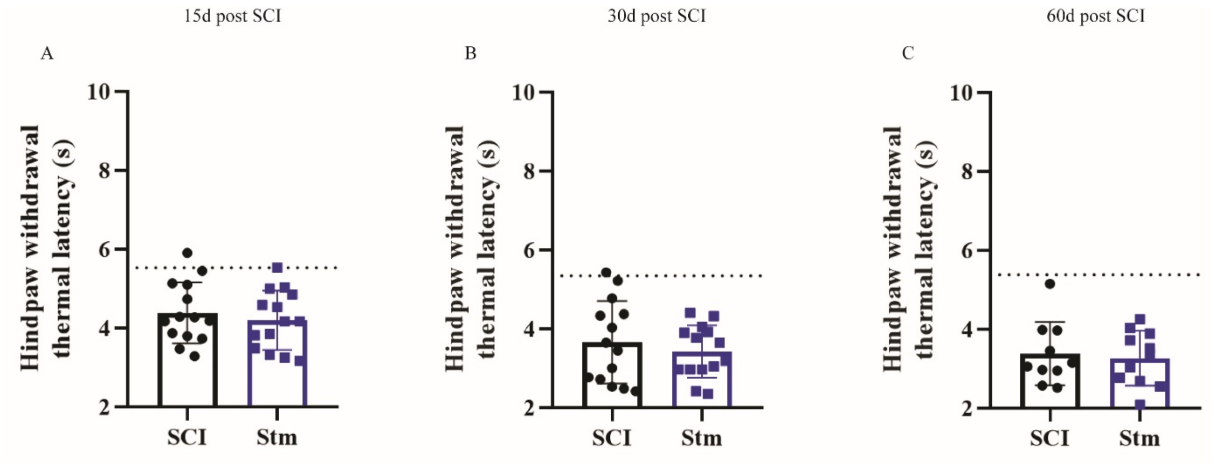

Supplement: Supplementary file 1 [file biomedicines-09-01827-s001.zip › biomedicines-1459079-supplementary.pdf]
